# Primary breast CD20-positive extranodal NK/T cell lymphoma with stomach involvement: a case report and literature review

**DOI:** 10.1186/s13000-021-01166-4

**Published:** 2021-11-08

**Authors:** Ying Zhang, Kuansong Wang, Qian Tan, Keda Yang, Dengshu Wu, Yajing Xu, Xielan Zhao, Zhiping Jiang

**Affiliations:** 1 Department of Hematology, Xiangya Hospital Central South University, 87 Xiangya Road, Changsha, 410008 People’s Republic of China; 2 Department of Pathology, Xiangya Hospital Central South University, 87 Xiangya Road, Changsha, 410008 People’s Republic of China

**Keywords:** Extranodal NK/T cell lymphoma, CD20 expression, Primary breast lymphoma, Case report, NGS

## Abstract

**Background:**

We present a unique case of primary breast CD20-positive extranodal NK/T cell lymphoma with stomach involvement in a young Chinese female patient.

**Case presentation:**

The patient presented with a mass in her right breast that rapidly increased in size over approximately 2 months. Upper gastrointestinal endoscopy showed a giant serpentine ulcer in the stomach. Biopsy was performed, and microscopic inspection revealed that the fibrous tissue was diffusely involved by medium to large abnormal lymphocytes. The cytoplasm was low to moderate. The tumor cells had irregular nuclei and inconspicuous nucleoli. The lymphoid cells were strongly immunoreactive to CD20, CD3, CD4, CD56, TIA-1, EBER, and Ki-67 (90%). Epstein-Barr virus genomes were also found in tumor cells by in situ hybridization. A whole-body positron emission tomography (PET)-CT scan revealed intense FDG uptake in the right breast and greater curvature of the stomach. Monoclonal rearrangements of the T cell receptor (TCR-γ) and immunoglobulin heavy chain (IgH) were identified by genetic analysis. Whole-genome next-generation sequencing was performed, and up to 12 gene mutations, including a frameshift mutation in exon 4 of the BCOR (G97Rfs*87; 44.3%) gene and a base substitution mutation (Q61H 17.6%) in exon 3 of the KRAS gene, were detected. Kyoto Encyclopedia of Genes and Genomes pathway analyses were performed using the database for annotation, visualization, and integrated discovery, which showed that rare primary breast CD20-positive extranodal NK/T cell lymphoma had a unique genetic background compared with diffuse large B cell lymphoma and extranodal NK/T cell lymphoma without CD20 expression. The patient received four cycles of the modified SMILE regimen. The second whole-body PET-CT scan revealed that the right breast mass was significantly smaller than before; additionally, FDG uptake in the stomach wall disappeared.

**Conclusions:**

Systemic examination, extensive immunohistochemistry, and molecular profiling are essential for an accurate diagnosis. More similar cases are required to clarify the biological pathways and even the potential molecular mechanisms of rare lymphomas, which may help direct further treatment.

## Background

Neoplasms of natural killer (NK) cells and NK-like T cells are uncommon hematological malignancies that occur more frequently in Asians than Caucasians. Strong CD20 expression in extranodal NK/T cell lymphoma (ENKTCL) is scarce, and only a few cases have been reported to date. Extranodal lymphoma, which includes primary and secondary involvement, accounts for less than 0.5% of all breast malignancies. Secondary involvement of the breast with lymphoma is more common than primary breast lymphoma (PBL). PBL is rare and accounts for less than 1% of all cases of non-Hodgkin’s lymphomas and approximately 0.1% of all cases of breast neoplasms. The most common pathological type of PBL is diffuse large B cell lymphoma with the activated B cell-like (ABC) type.

CD20, also named MS4A1, is a 35-kDa transmembrane protein regarded as a specific marker of B cell lineage commitment [1]. It plays a vital role in cell differentiation, signal transduction, and cell cycle regulation. It also serves as a target for therapeutic monoclonal antibodies in treating B cell lymphomas and leukemia [2]. As a result, CD20 is essential for the diagnosis and treatment of B- cell lymphoma. However, CD20 expression on NK cells and NK-like T cells of lymphomas has rarely been found, which may result in potential diagnostic dilemmas.

Huang et al. [3] summarized 18 CD20-positive ENKTCL reported to date, but no breast involvement was found. Next-generation sequencing (NGS) was also performed in this research but only covered the target genes MS4A1 (CD20), CD3E (CD3), and PAX5 instead of total genomic DNA.

In this paper, we describe a unique case of PBL, which was diagnosed as ENKTCL with CD20 strong expression. Monoclonal rearrangements of the T cell receptor (TCR) and immunoglobulin heavy chain (IgH) identified. Whole-genome NGS was also performed, and the mutation background was compared with those of diffuse large B cell lymphoma (DLBCL) and ENKTCL without CD20 expression. To our knowledge, PBL diagnosed as CD20-positive ENKTCL has not been previously reported. We also compared the mutation background data with the data of DLBCL patients and ENKTCL patients without CD20 expression by Kyoto Encyclopedia of Genes and Genomes (KEGG) pathway analysis. We also reviewed other cases in the literature.

## Case presentation

### Clinical presentation and management

**Fig. 1:**
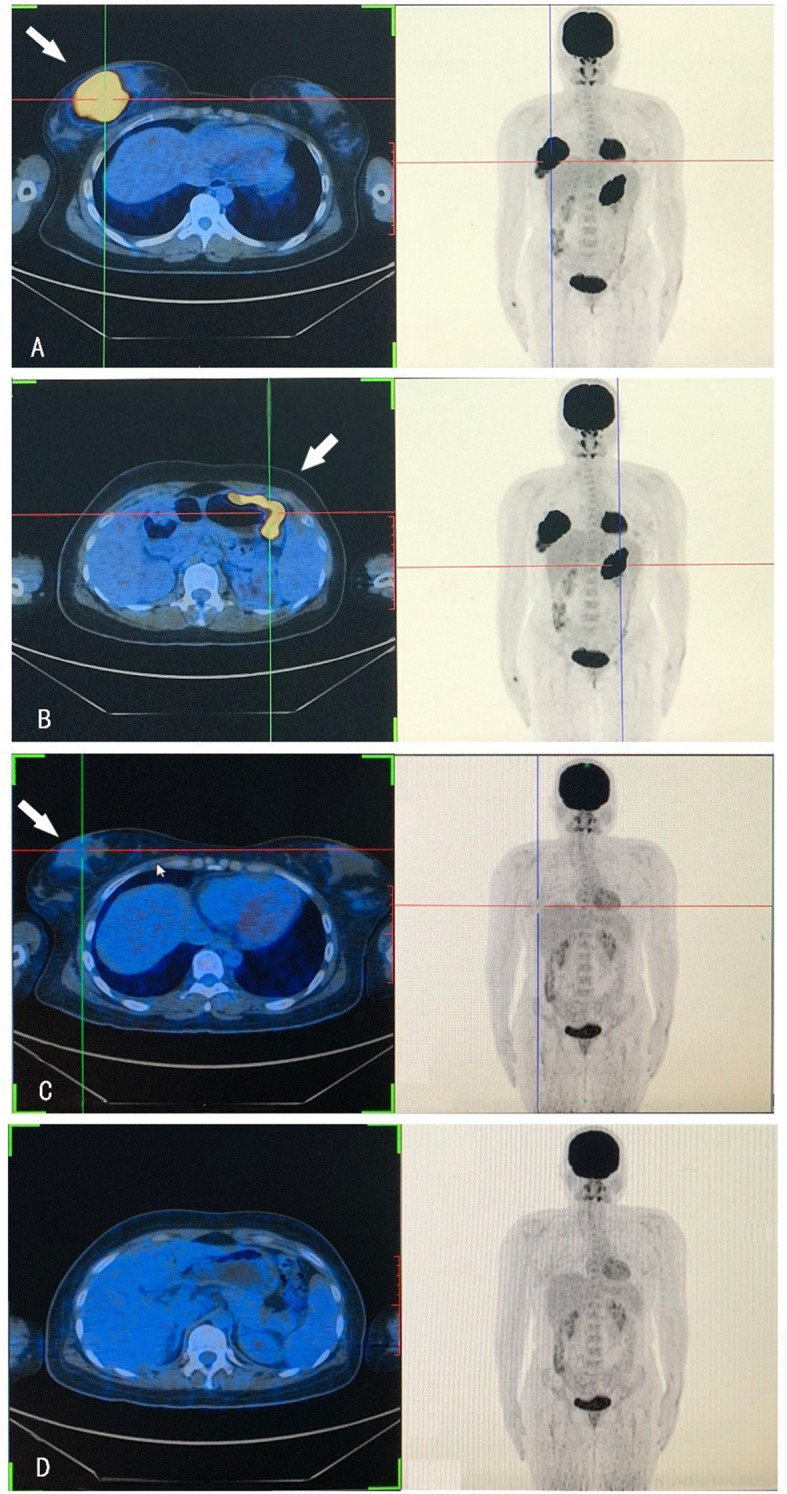
A whole-body positron emission tomography (PET)-CT scan revealed intense FDG uptake in the right breast **(A)** and greater curvature of the stomach **(B).** After four cycles of modified SMILE regimen, the right breast mass was significantly smaller than before (C). FDG uptake in the stomach wall disappeared (D)

**Fig. 2:**
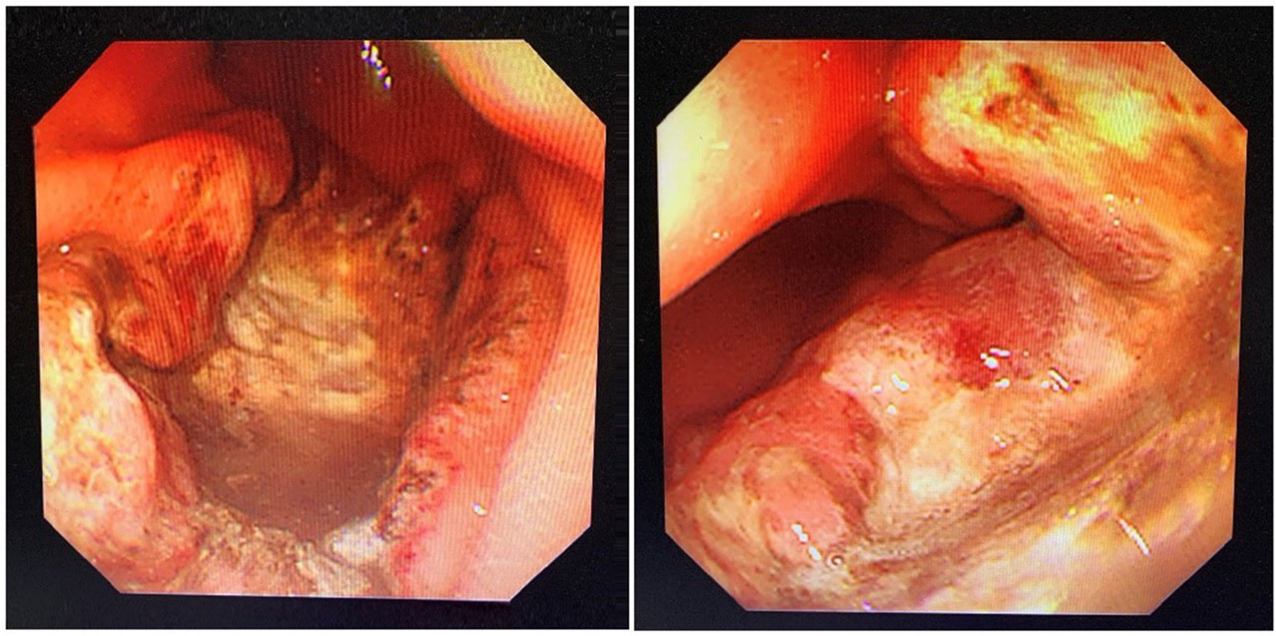
Upper gastrointestinal endoscopy showed a giant serpentine ulcer in the stomach

A 30-year-old woman had presented with a rapidly growing mass in her right breast for approximately 2 months. Ultrasonography of the right breast showed a 34-mm anterior-posterior (AP) hypoechoic area with partial duct dilatation. In the outpatient clinic, she underwent a thick needle biopsy of the right breast. The initial pathology report indicated a malignant tumor of the lymphoid hematopoietic system; such tumors tend to be of B cell origin. However, the pathologic material was insufficient for final histologic categorization. As a result, the patient underwent resection of part of the right breast mass and was diagnosed with ENKTCL with aberrant CD20 expression. A whole-body positron emission tomography (PET)-CT scan was recommended to review FDG uptake. PET-CT revealed intense FDG uptake in the right breast (SUVmax 28.3) (Fig. 1A) and greater curvature of the stomach (SUVmax 19.2) (Fig. 1B). However, PET-CT showed normal uptake of FDG in the nose, which is different from the clinic features of ENKTCL. Upper gastrointestinal endoscopy showed a giant serpentine ulcer in the stomach (Fig. 2). Biopsies of the breast and stomach were performed. Bone marrow aspirate, bone marrow biopsy, and flow cytometry revealed no malignant cells. Cytogenetic analysis revealed a normal female karyotype.

After diagnosis, the patient received four cycles of a modified SMILE regimen, including methotrexate, dexamethasone, ifosfamide, etoposide, and pegaspargase. A second whole-body PET-CT scan revealed that the right breast mass was significantly smaller than before (Fig. 1C), and FDG uptake in the stomach wall had disappeared (Fig. 1D). Subsequently, the patient refused further treatment.

## Material and methods

### Pathology and immunohistochemistry

Following routine procedures, the resected breast and gastric biopsy tissues were fixed in 10% neutral buffered formalin and embedded in paraffin. For hematoxylin and eosin (H&E) staining, four-micrometer-thick sections were sliced using a Leica Autostainer (Histocore SPECTRS STCV, Germany). Immunohistochemistry was performed using the streptavidin–biotin complex immunoperoxidase technique. The antibodies used in this study were CD3, CD4, CD5, CD8, CD10, CD20, CD23, CD56, CD79a, CD99, PAX-5, Bcl-6, Bcl-2, UM1, CyclinD1, SOX11, TdT, CK-Pan, TIA-1 and Ki-67. All of these antibodies were purchased from Dako Cytomation (USA). Slides were routinely dewaxed, rehydrated and then treated in the microwave with ten mmol citrate buffer (PH 6.0). Then, after incubation with primary antibodies, the slides were treated with the ChemMate Envision/HRP Kit for 30 min at room temperature, followed by development with diaminobenzidine for visualization.

In situ hybridization for Epstein-Barr virus-encoded RNA (EBER) was performed to determine the status of Epstein-Barr virus (EBV) infection on paraffin-embedded tissues. The EBER detection kit was purchased from Dako (Glostrup, Denmark). Appropriate positive and negative controls were used for all stains. The detection process was conducted according to the manufacturer’s instructions.

Genomic DNA was extracted from tissue using DNA extraction and purification kit (Promega, Madison, WI, USA). T cell receptor (TCR-β, γ, δ) and immunoglobulin gene rearrangement (IgH, K, L) assays were performed. Each PCR study was carried out in duplicate and included negative, positive, and blank controls. β-actin was amplified as an internal control. The PCR products were analyzed by capillary electrophoresis using the ABI HITACHI 3500 Genetic Analyzer (Applied Biosystems, CA, USA).

### Next-generation sequencing assay

NGS was also performed to investigate the genetic background. Formalin-fixed paraffin-embedded (FFPE) tissue samples were collected from patients. Peripheral blood from the same patient was collected as the germline control.

Genomic DNA was extracted from FFPE tissue and peripheral blood using the QIAamp DNA FFPE Tissue Kit (Qiagen, Hilden, Germany) and QIAamp DNA Blood Mini Kit (Qiagen), respectively. DNA concentrations were measured using a Qubit fluorometer and Qubit dsDNA HS (High-sensitivity) Assay Kit (Invitrogen, Carlsbad, CA, USA). The distribution of plasma DNA was assessed using an Agilent 2100 BioAnalyzer and the DNA HS Kit (Agilent Technologies, Santa Clara, CA, USA). DNA was sheared into 200–250-bp fragments with a Covaris S2 Ultrasonicator (Covaris, Woburn, MA, USA). Subsequently, hybridization with capture probe baits, hybrid selection with magnetic beads, and polymerase-chain-reaction amplification were performed. Target region capture was performed using 1–2 μg of DNA with a custom SeqCap EZ Library (Roche NimbleGen, Madison, WI, USA) according to the manufacturer’s protocol. The capture probe was designed based on ~ 2.1-Mb genomic regions of 619 genes that are frequently mutated in lymphoma and hematologic malignancies. Capture hybridization was carried out according to the manufacturer’s protocol. Following hybrid selection, the captured DNA fragments were amplified and pooled to generate multiplex libraries. Paired-end sequencing was performed with Illumina 2 × 75-bp paired-end reads on an Illumina HiSeq 3000 instrument according to the manufacturer’s recommendations using the TruSeq PE Cluster Generation Kit v3 and TruSeq SBS Kit v3 (Illumina, San Diego, CA, USA), which was conducted by a commercial vendor (Geneplus-Beijing, China).

### Mutation calling

After removing sequencing reads containing adaptor sequences and low-quality reads, the remaining high-quality paired-end reads were aligned to the human reference genome (build hg19) using BWA (0.7.12-r1039) [4]. MuTect2 (3.4–46-gbc02625) [5] was employed to call somatic small insertions and deletions (InDels) and single nucleotide variants (SNVs). Contra (2.0.8) [6] was used to detect copy number variations, and an in-house algorithm was used to identify split-read and discordant-read pairs to identify structural variants.

### KEGG analysis

To compare the mutation backgrounds of ENKTCL patients without CD20 expression and DLBCL patients, we searched the raw data of two published papers, and the three groups of data were analyzed by R version 3.6.1. KEGG enrichment analysis was performed by a cluster profiler. Pathways with *p*-values less than 0.05 were retained to construct a bubble chart. The X-axis indicates the “rich factor” represented by the ratio of co-expressed gene numbers to the total gene numbers of each pathway, and the left Y-axis represents the KEGG pathways. Low *p*-values are shown in red, and high *p-*values are shown in green on the right. The area in the circle represents the co-expressed gene number.

## Results

### Pathological findings

**Fig. 3:**
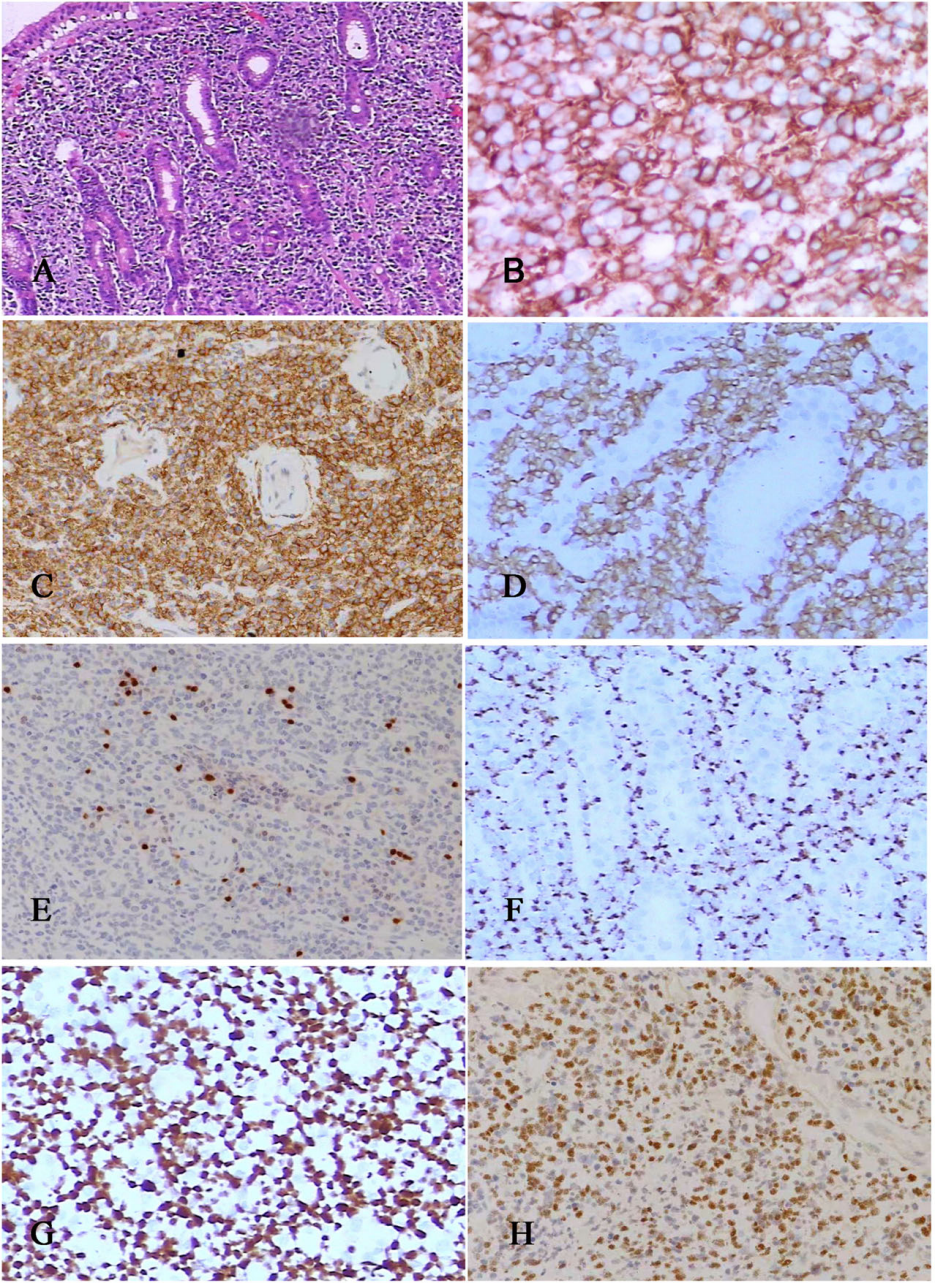
Photomicrographs of the resected breast. Under the microscope, the mass showed the fibrous tissue consisted of medium to large abnormal lymphocytes, which were diffusely distributed. The tumor cells had irregular nuclei and inconspicuous nucleoli (A). Immunohistochemical analysis of the breast showed tumor cells were positive for CD20 (B), CD56 (C), CD3 (D), PAX-5 (E), TIA-1 (F). (G) The ki-67 positive rate was 90%. (H) Tumor cells were also positive for Epstein-Barr virus small-encoded RNA (EBER) by in situ hybridization (A, HE staining with original magnification × 100; B-F, immunohistochemical staining with original magnification × 200; H, in situ hybridization for EBER with original magnification × 200)

**Fig. 4:**
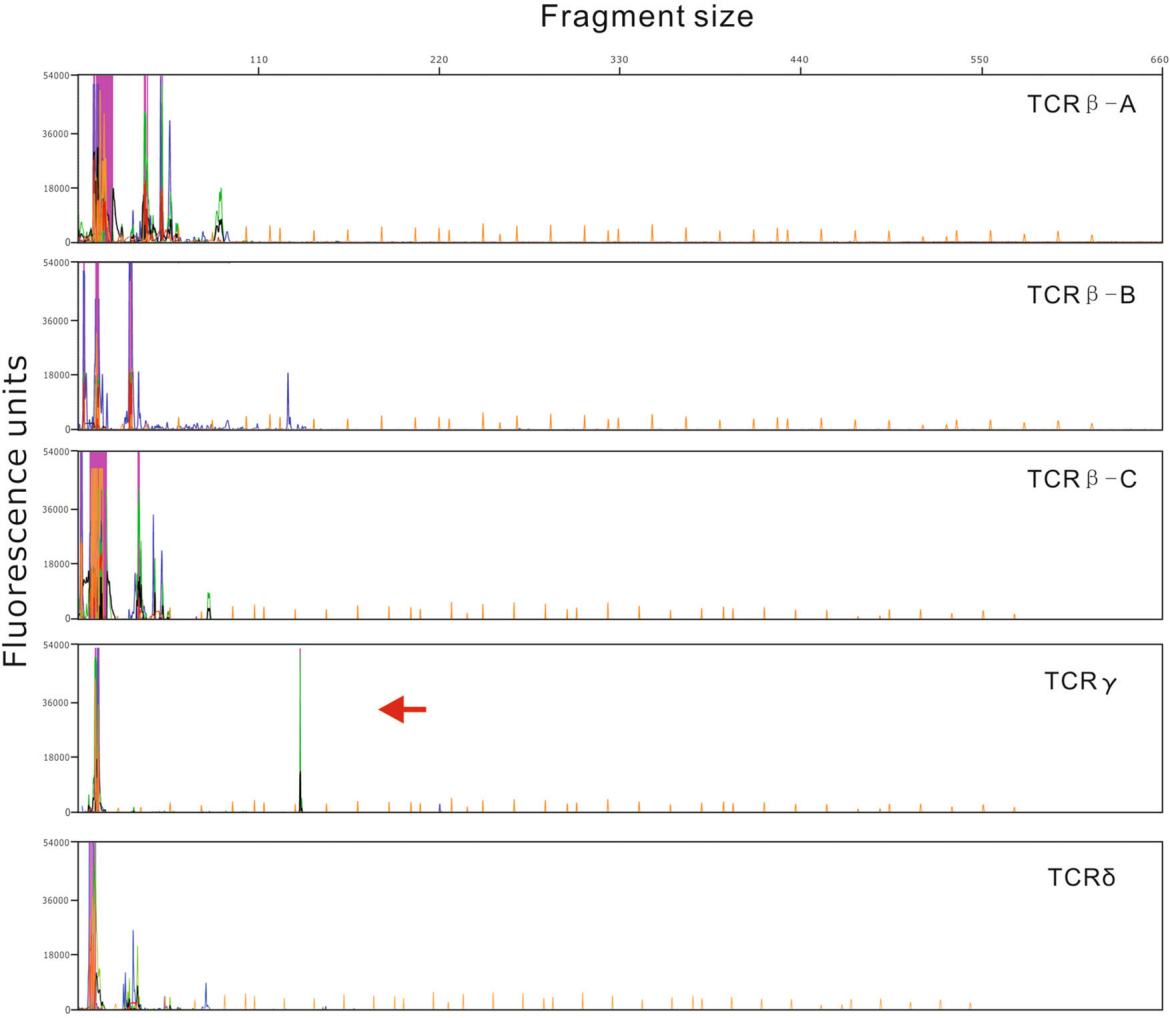
Molecular testing for gene rearrangement of TCR. The tumor demonstrates a monoclonal peak for TCR-γ

**Fig. 5:**
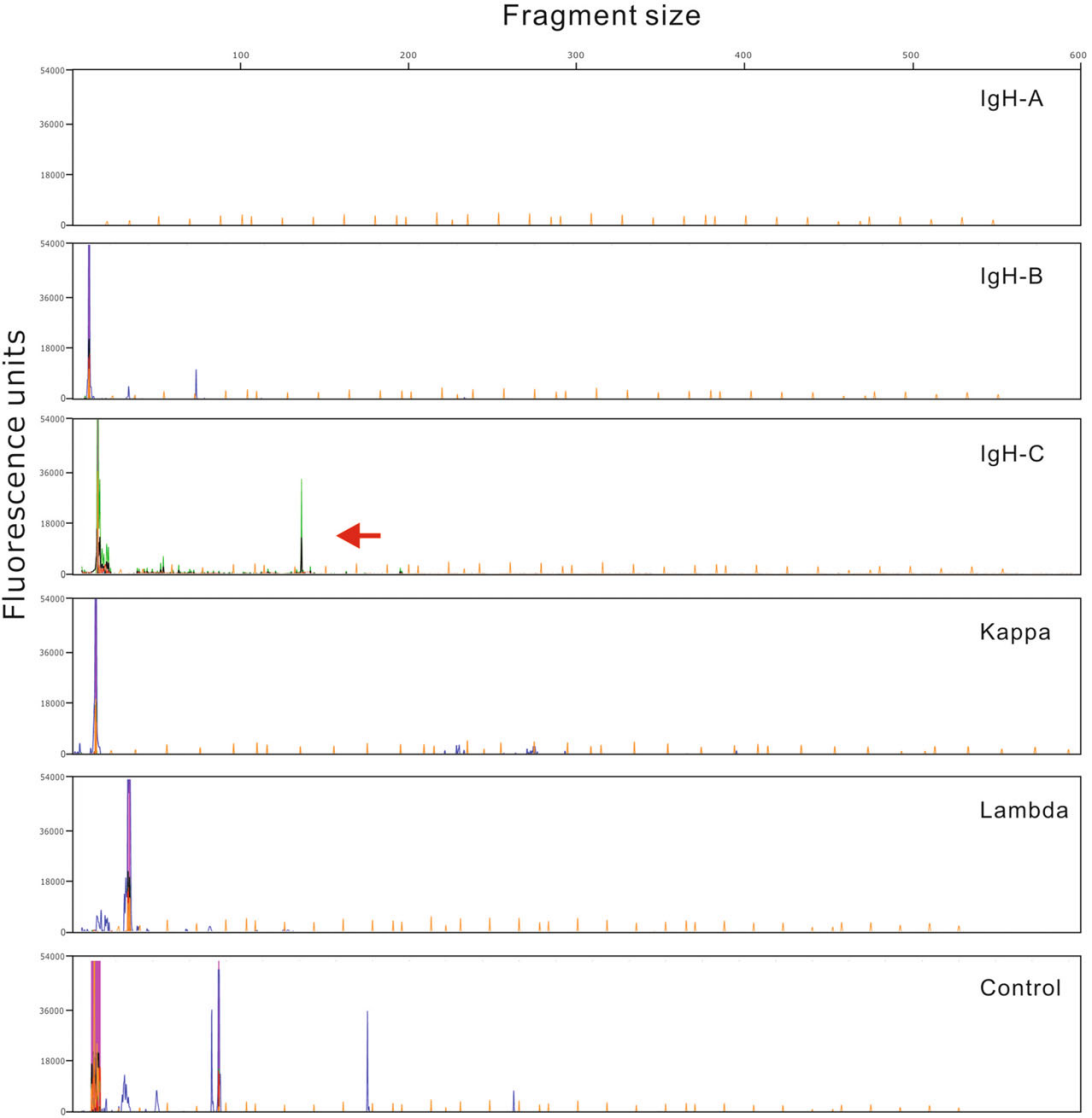
Molecular testing for gene rearrangement of IgH. The tumor demonstrates a monoclonal peak for IgH-C

Pathological sections showed that the fibrous tissue consisted of diffuse medium to large abnormal lymphocytes. The cytoplasm was low to moderate. The tumor cells had irregular nuclei and inconspicuous nucleoli (Fig. 3A). Immunohistochemical studies showed that the tumor cells were strongly positive for CD20 (Fig. 3B). The cells were also positive for the NK cell marker CD56 (Fig. 3C) and the T cell markers CD3 (Fig. 3D) and CD4; the cells were negative for the T cell markers CD5 and CD8. Other B cell markers, including CD79a, CD10, CD23, OCT-2, Bob-1, and PAX-5 (Fig. 3E), were negative. The tumor cells were also positive for the cytotoxic marker TIA-1 (Fig. 3F). Ki67 was positive in more than 90% of cells (Fig. 3G). In situ hybridization for EBER was markedly positive (Fig. 3H). Detection of clonally rearranged Ig and TCR genes revealed that the TCR-γ gene (Fig. 4) and IgH gene (Fig. 5) both showed monoclonal rearrangement.

### NGS analysis

A frameshift mutation in exon 4 of the BCOR (G97Rfs*87; 44.3%) gene and a base substitution mutation (Q61H 17.6%) in exon 3 of the KRAS gene were detected in this patient by NGS. No mutations in the target genes MS4A1 (CD20), CD3E (CD3), and PAX5 were detected in the analysis.

### KEGG analysis

**Fig. 6:**
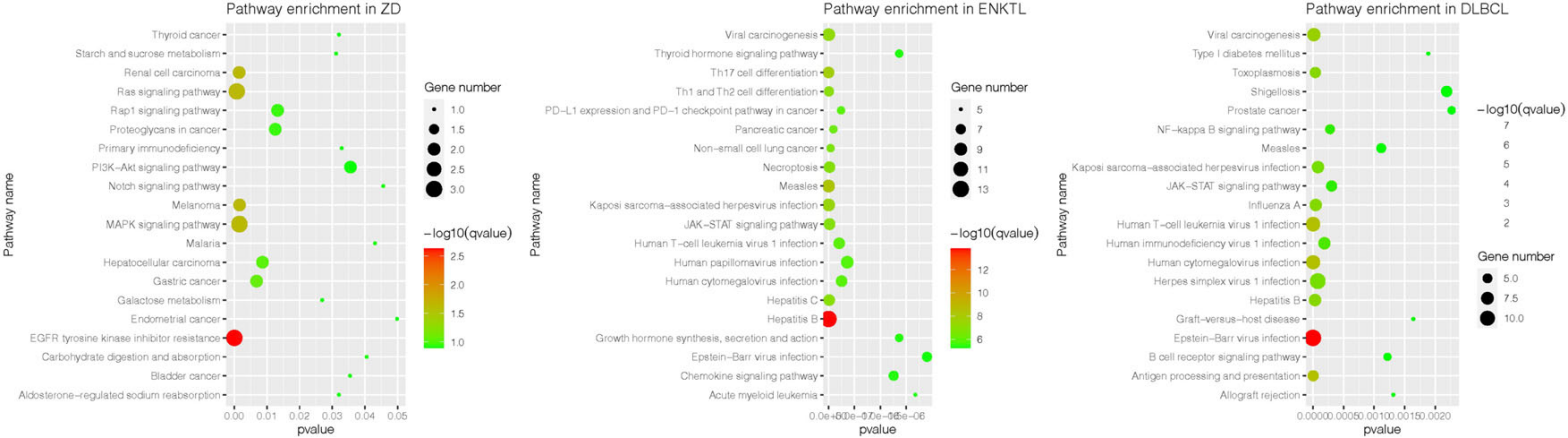
The cluster Profiler of KEGG enrichment analysis (The size of the bubble represents the gene numbers, and depth of color represents enrichment *p*-value. The nearer to red represents a smaller *p-*value. We found they were three different enriched signaling pathways respectively)

The KEGG analysis results are shown in Fig. 6. The results of the KEGG pathway enrichment analysis showed that 619 genes were enriched in 40 pathways. EGFR tyrosine kinase inhibitor resistance, hepatitis B, and EBV infection were the most enriched signaling pathways in this patient, ENKTCL patients without CD20 expression and DLBCL patients.

## Discussion and conclusion

This paper, reports a case of ENKTCL expressing CD20, CD99, CD3, CD4, CD56, and EBER with breast and stomach involvement and simultaneous monoclonal rearrangement of the IgH and TCR genes. This case underlines the importance of precise and complete immunohistochemical analysis to accurately diagnose hematological malignancies, including lymphoma and leukemia.

The typical immunophenotype of ENKTCL includes positivity for CD3 (or CD2), CD56, cytotoxic markers (such as granzyme B, TIAI, and perforin), and EBV [7, 8]. Schwartz et al. characterized 84 cases of ENKTCL concerning the expression of immunohistochemical markers using a tissue microarray and found that CD20 was not expressed in any case [9]. In our case, CD20 was overexpressed, while the other B cell-associated antigens were all negative. The most common site of ENKTCL is the nasal area, while 20% of cases present outside the nasal area [10], which is associated with a poor prognosis. With the wide application of PET/CT for lymphomas, an increasing number of non-nasal cases have been found to have occult nasal primary lesions [11]. In our case, occult nasal primary lesions were excluded by PET/CT. As a result, based on the clinical presentation, immunophenotype, and PET/CT results, a diagnosis of ENKTCL in a non-nasal area with abnormal CD20 expression was established.

We found that only eighteen cases of CD20-positive NK/T cell lymphoma have been reported in the literature [3]. However, this is the first case of ENKTCL with breast and stomach involvement. The initial presenting symptoms of primary gastrointestinal T/NK cell lymphoma (GI-TNKL) are usually gastrointestinal bleeding, abdominal pain, and epigastric soreness, but our patient did not show these symptoms. Mutations in GI-TNKLs were enriched in three different biological pathways: the voltage-gated potassium (Kv+) channel, the JAK-STAT pathway, and the immunoglobulin superfamily, which differs from observations in non-GI NK/T cell lymphoma [12]. The NGS results of our case are not similar to the characteristics of GI-TNKLs bearing BCOR and KRAS mutations, which are two typical mutations of ENKTCL. The results implied that the malignancy in our case was more likely PBL than primary gastrointestinal lymphoma.

PBL is a relatively rare extranodal lymphoma subtype, accounting for 0.5% of all breast malignancies, less than 3% of all extranodal lymphomas, and approximately 1% of all non-Hodgkin lymphomas (NHLs) [13–15]. DLBCL (56-84%), mantle cell lymphoma (9-28%), follicular lymphoma (10-19%), and Burkitt lymphoma (< 6%) are the most common pathological types of PBL, and other pathological types, such as anaplastic large cell lymphoma, peripheral T cell lymphoma, small lymphocytic lymphoma, lymphoplasmacytic lymphoma, mantle cell lymphoma, and Hodgkin lymphoma, are relatively rare [16]. Among PBLs, most are of the B cell lineage, with only rare cases showing a T cell immunophenotype, while NK/T cell lymphoma originating from the breast is exceedingly rare, with only five cases reported to date [17–21]. Two patients were diagnosed with nasal-type ENKTCL involving the breast and nasopharynx [17, 18]. Two cases were associated with breast implants placed for cosmetic reasons [19, 20]. One case involved the breast as part of disseminated disease in an immunosuppressed woman who had undergone heart transplantation 5 years prior [21].

Huang et al. [3] summarized 18 CD20-expressing ENKTCL cases, which was the only reported case series (Table 1). The 18 cases involved 3 females and 15 males with a median age of 58 years (range 25 to 81 years); 16 patients were Chinese, 1 was Japanese, and 1 was Caucasian. Three patients had involvement in more than 1 extranodal site. The primary tumors were located in the nasal cavity (5), testis (3), skin and soft tissue (4), scrotum (2), thenar eminence (1), stomach (2), chest wall (1), leg (1), back (1), and central nervous system (1). All T cell markers were negative, and only two of 18 cases showed monoclonal rearrangement of the IgH gene, while our case showed monoclonal peaks for both the IgH and TCR genes (Figs. 4, 5). In the study of Huang et al., 7 of 18 cases were successfully analyzed with NGS but only for the target genes MS4A1 (CD20), CD3E (CD3), and PAX5. However, these mutations were not significantly functional, as predicted by SIFT (*p* > 0.05). In our case, 619 genes were analyzed with NGS, and no MS4A1 (CD20), CD3E (CD3), or PAX5 gene mutations were found. Sixteen other gene mutations were detected (Table 2). To our knowledge, apart from the ASXL1, BCORL1, MGAM (c.136C > T), and KRAS genes, the remaining genes showed novel mutations that were not recorded in the COSMIC (Catalog of Somatic Mutations in Cancer) database and MSK (Memorial Sloan Kettering) database. By consulting the literature, we found that gene expression, in this case, differed from that in ENKTCL and DLBCL cases [22, 23]. To further confirm this finding, we analyzed signaling pathways in these three diseases using KEGG analysis. The results showed that 619 genes were enriched in 40 pathways (Fig. 6). Obviously, this patient’s signaling pathway profile was completely different from those observed in the other two categories. EGFR tyrosine kinase inhibitor resistance, hepatitis B virus, and EBV infection were the most enriched signaling pathways in this patient, ENKTCL and DLBCL patients, respectively. As a result, we hypothesize that primary breast CD20-positive ENKTCL might be completely a new lymphoma subtype due to the possibility of unique genetic background. However, this is the only case expressing this type of clinic features. Further investigation including more cases is needed.

**Table 1:** Clinicopathologic features of NK/T-cell lymphoma patients with aberrant expression of CD20 in present and previous literature review

No.	Author (yr.)	Age/Gender (Ethnicity)	Primary site	Immunophenotype	BCR/TCR	Treatment	Follow-up (months)
1	Present study	30/F (Chinese)	Breast,stomach	CD20+++, CD3 + , CD4 + , CD56+++, TIA-1+, EBER(+), Ki67(90%)	+/+	CT (SMILE)	11,Alive
2	Huang,et al. (2020)	67/M (Chinese)	Testis	CD20 + , CD3 + , CD2 + , CD7 + , CD56 + , TIA-1+,Gran B+, Perforin+,EBER(+),Ki67(90%)	+/−	Or + CT (P-GEMOX)	4, Alive
3	Huang,et al. (2020)	41/F (Chinese)	Nasal cavity	CD20 + , CD3 + , CD56 + , TIA-1+,Gran B+, Perforin+, EBER(+),Ki67(70%)	NA/NA	CT (EPOCH)	NA
4	Huang,et al. (2020)	37/F (Chinese)	Skin	CD20 + , CD3 + , CD2 + , CD56 + , TIA-1+,Gran B+, EBER(+), Ki67(60%)	−/−	CT (P-GEMOX)	9,DOD
5	Huang,et al. (2020)	62/M (Chinese)	Nasal cavity	CD20 + , CD3 + , CD2 + , CD7p + , CD56 + , TIA-1+,Gran B+, Perforin+,EBER(+),Ki67(60%)	NA/NA	CT (AspaMetDex + P-GEMOX)	18,DOD
6	Huang,et al. (2020)	79/M (Chinese)	Testis	CD20 + , CD3 + , CD2p + , TIA-1+, EBER(+), Ki67(80%)	−/−	Or	0.5,DOD
7	Huang,et al. (2020)	67/M (Chinese)	Nasal cavity	CD20 + , CD3 + , CD7 + , CD56 + , TIA-1+,Gran B+, EBER(+),Ki67(80%)	−/−	CT (P-GEMOX)	4,Alive
8	Huang,et al. (2020)	29/M (Chinese)	Skin,scrotum	CD20 + , CD3 + , CD2 + , CD56+ TIA-1+,Gran B+, Perforin+, EBER(+),Ki67(80%)	−/−	CT(P-GEMOX +GVD+ Pembrolizumab+PDL1+ Benzamine +Daratumumab +PD-1)	39,Alive
9	Huang,et al. (2020)	37/M (Chinese)	Testis	CD20 + , CD3 + , CD7 + , CD56 + , CD79a F+, TIA-1+, EBER(+),Ki67(75%)	NA/NA	Or + CT (Not specific)	NA
10	Huang,et al. (2020)	60/M (Chinese)	Soft tissue, LN Nasal cavity, scrotum	CD20 + , CD3 + , CD2 + , CD7 + , CD56 + , TIA-1+,Gran B+, Perforin+,EBER(+),Ki67(60%)	−/−	CT (Not specific)	2,Alive
11	Huang,et al. (2020)	56/M (Chinese)	Skin	CD20 + , CD3 + , CD2 + , CD7 + , CD56 + , TIA-1+,Gran B+, Perforin+,EBER(+),Ki67(50%)	−/−	CT (GEMOX-L)	2, Alive
12	Huang,et al. (2020)	81/M (Chinese)	Nasal cavity, scrotum	CD20 + , CD3 + , CD7 + , CD56 + , TIA-1+, EBER(+), Ki67(90%)	NA/NA	CT (P-GEMOX)	3, Alive
13	Li D,et al. (2017)	27/M (Chinese)	Left cerebellum	CD20 + , CD3 + , CD2 + , Gran B+, EBER(+),Ki67(85%)	+/−	RT + steroid therapy	3,DOD
14	Huang YH,et al. (2015)	48/M (Taiwancsc)	Stomach	CD20 + , CD3+, CD56 + , TIA-1+,Gran B+, EBER(+), Ki67(90%)	NA/−	C T (V I P, S M I L E)	8,DOD
15	Tsai YC,et al. (2015)	32/M (Taiwancsc)	Leg, back, LN	CD20 + , CD3 + , CD2 F + , CD56 + , TIA-1+,EBER(+)	NA/NA	CT (DICE)	6,Alive
16	Jiang QP,et al. (2012)	78/F (Chinese)	Nasal cavity	CD20 + , CD3 + , CD2 + , CD56 + , TIA-1+,Gran B+, EBER(+), Ki67(60%)	−/−	Observation without treatment	6,Alive
17	Gill HS,et al. (2010)	25/M (Chinese)	Chest wall	CD20+, CD2 + , CD56 + , TIA-1+, EBER(+),Ki67(90%)	−/−	NA	NA
18	Kobold S,et al. (2009)	69/M (Caucasian)	Stomach	CD20 + , CD3 + , CD2 + , CD56 + , TIA-1+,Gran B+, Perforin+,	−/−	CT (CHOP)	1,DOD
19	Ando J,et al. (2008)	71/M (Japanese)	Thenar	CD20 + , CD3 + , CD2 + , CD56 + , TIA-1+,Gran B+, EBER(+)	−/−	CT (CHOP, ESHAP, and L-asparaginase)	6,DOD

*M* male, *F* female, *DOD* die of disease, *Or* orchiectomy, *CT* chemotherapy, *SMILE* dexamethasone,methotrexate, ifosfamidpegaspargase, and etoposide, *P-GEMOX* pegaspargase, gemcitabine and oxaliplatin, *GVD* gemcitabine, doxorubicin liposomes and vinorelbine, *GEMOX-L* gemcitabine and oxaliplatin and L-asparaginase, *EPOCH* VP-16, epirubicin/adriamycin, vincristine, cyclophosphamide, and prednisone, *AspaMetDex* pegaspargase, methotrexate, and dexamethasone, *CHOP* cyclophosphamide, doxorubicin, vincristine, and prednisone, *ESHAP* etoposide, methylprednisolone, cytarabine, cisplatin, *DICE* dexamethasone, etoposide, ifosfamide, and cisplatin, *RT* radiotherapy. *VIP* VP-16, ifosfamide, and cisplatin;

**Table 2:** Gene mutations detected by NGS

Gene Symbol	cHGVS	pHGVS	Function	Transcript	ExIn_ID	AF
BCOR	c.288_292delGGGCT	p.G97Rfs*87	frameshift	NM_001123385.1	EX4	0.44281
ASXL1	c.2485C > T	p.Q829*	nonsense	NM_015338.5	EX13E	0.5611
NCOR2	c.5469_5470insCGGC	p.S1824Rfs*194	frameshift	NM_001206654.1	EX38	0.047529
FAT3	c.4278G > T	p.R1426S	missense	NM_001008781.2	EX6	0.325153
CIITA	c.2029G > T	p.E677*	nonsense	NM_000246.3	EX11	0.357877
HGF	c.1520G > A	p.W507*	nonsense	NM_000601.4	EX13	0.26652
MGA	c.2922GCA[4 > 3]	p.Q974[8 > 7]	cds-del	NM_001164273.1	EX8	–
CIITA	c.1907C > T	p.T636M	missense	NM_000246.3	EX11	0.42402
LRP1B	c.4163G > A	p.R1388K	missense	NM_018557.2	EX25	–
BCORL1	c.1624G > A	p.D542N	missense	NM_021946.4	EX3	–
MGAM	c.160C > T	p.P54S	missense	NM_004668.2	EX3	–
MGAM	c.136C > T	p.P46S	missense	NM_004668.2	EX3	–
KRAS	c.183A > C	p.Q61H	missense	NM_033360.2	EX3	0.175824
DIS3	c.1982C > T	p.S661F	missense	NM_014953.3	EX16	0.125731
NF1			gain	NM_001042492.2	17q11.2	2.10
SUZ12			gain	NM_015355.2	17q11.2	2.07

Mutations in the BCOR and KRAS genes were detected in our patient by NGS. This frameshift mutation in BCOR results in the substitution of glycine to arginine at the 97th position in the BCOR gene coding region, and transcription termination at the 183rd position may produce a protein with impaired function. BCOR interacts with BCL6 via the POZ domain and plays a critical role in the formation of germinal centers and apoptosis. BCOR alterations have been reported to tend to emerge more frequently in malignancies associated with EBV infections and often occur in the form of loss-of-function mutations in exon 4. Thus, EBV infection, together with BCOR mutations, can be speculated to lead to ENKTCL. Multiple studies in Southeast Asia showed that BCOR, TP53, STAT3, and DDX3X were the most frequently mutated genes in nasal-type ENKTCL, and BCOR mutations were detected in approximately 5.7 to 32% of cases [24–26]. However, this mutation has never been reported previously and has not been recorded in the COSMIC and MSK databases. KRAS mutations were reportedly detected in 5.9% of ENKTCL patients [27]. The KRAS mutation Q61H could upregulate the function of KRAS and might play an important role in cancer genesis, and targeting KRAS in cancer therapy should hopefully facilitate the development of new strategies [28].

Simultaneous monoclonal rearrangement of the IgH and TCR genes creates a diagnostic dilemma. Generally, TCR rearrangement is associated with T cell proliferation and IgH rearrangement with B cell proliferation, but this may not always be true. Observations of “lineage infidelity,” where B cells express TCR markers, T cells express IgH markers, or B and T cells express markers of both cell lineages due to gene rearrangements, have been reported in the literature [29, 30]. Huang et al. included only two cases with monoclonal rearrangement of the IgH gene.

ENKTCL is associated with a poor clinical outcome. The 2-year OS of CD20-positive ENKTCL patients has been reported to be 24%, with a median survival time of only 11.5 months [3]. Whether CD20 can be used as a target for CD20-positive ENKTCL therapy is unclear. None of the 18 cases of CD20-positive ENKTCL were treated with rituximab, which has been widely used for CD20-positive B cell lymphomas. Whether rituximab offers a survival advantage in CD20-positive ENKTCL warrants further investigation. Conventional chemotherapy with anthracycline-containing regimens such as CHOP [31] is mainly ineffective for ENKTCL due to the P-glycoprotein in NK lymphoma cells [32]. L-asparaginase-based regimens such as SMILE, AspaMetDex, and P-Gemox are recommended for ENKTCL treatment by NCCN guidelines due to a lack of asparagine synthase in this malignancy, which augments the sensitivity of ENKTCL to L-asparaginase [33]. The SMILE regimen used in our patient yielded favorable preliminary results. However, the long-term clinical outcome requires further investigation.

In conclusion, we report a case of CD20-positive ENKTCL with breast and stomach involvement and simultaneous monoclonal rearrangement of the IgH and TCR genes. Systemic examination, extensive immunohistochemistry, and molecular profiling are essential for an accurate diagnosis. More similar cases are required to clarify the biological pathways and even the potential molecular mechanisms of rare lymphomas, which may help direct further treatment.

## Data Availability

All data generated or analyzed during this study are included in this published article.

## Abbreviations

NK: Natural killer
ENKTCL: Extranodal NK/T cell lymphoma
PBL: Primary breast lymphoma
TCR: T cell receptor
IgH: Immunoglobulin heavy chain
EBER: Epstein-Barr virus-encoded RNA
EBV: Epstein-Barr virus
GI-TNKL: Primary gastrointestinal T/NK cell lymphoma
DLBCL: Diffuse large B cell lymphoma
KEGG: Kyoto Encyclopedia of Genes and Genomes
